# Depression, Anxiety, Resilience and Coping Pre and Post Kidney Transplantation – Initial Findings from the *Psychiatric Impairments* in *Kidney Transplantation* (*PI-KT*)-Study

**DOI:** 10.1371/journal.pone.0140706

**Published:** 2015-11-11

**Authors:** Helge H. Müller, Matthias Englbrecht, Michael S. Wiesener, Stephanie Titze, Katharina Heller, Teja W. Groemer, Georg Schett, Kai-Uwe Eckardt, Johannes Kornhuber, Juan Manuel Maler

**Affiliations:** 1 Department of Psychiatry and Psychotherapy, Friedrich-Alexander Universität Erlangen-Nürnberg (FAU), Erlangen, Germany; 2 Department of Internal Medicine 3—Rheumatology and Immunology, Friedrich-Alexander University Erlangen-Nürnberg (FAU), Erlangen, Germany; 3 Department of Internal Medicine 4—Nephrology and Hypertension, Friedrich-Alexander Universität Erlangen-Nürnberg (FAU), Erlangen, Germany; UNIFESP Federal University of São Paulo, BRAZIL

## Abstract

**Purpose:**

Depression/anxiety, impaired Health-Related Quality of Life (HRQoL) and coping and resilience structures, are associated with increased mortality/poor outcome in chronic kidney disease (CKD) patients before (CKD/pre-KT) and after kidney (CKD-T) transplantation. Less is known about prevalence rates of psychiatric symptoms and impaired HRQoL of non-transplanted compared with transplanted patients.

**Methods:**

In a cross-sectional study comparing 101 CKD/pre-KT patients with 151 cadaveric-transplanted (CKD-T) patients, we examined prevalence of depression/anxiety (HADS questionnaire) and coping, resilience and HRQoL (SF-12, Resilience-Scale and FKV-questionnaire).

**Results:**

The prevalence of both depressive and anxiety symptoms was not significantly different between different pre-/and CKD-T patient groups. In CKD-T no significant relations of coping strategies with kidney function were identified. Furthermore, the Resilience Scales for acceptance and competence did not suggest any differences between the CKD/pre-KT and CKD-T subgroup. In the CKD/pre-KT patients, significant correlations were identified between the acceptance subscale and partnership, as well as between the competence subscale and older age/partnership.

**Conclusions:**

Both the CKD/pre-KT and CKD-T patients exhibited notable impairments in the HRQoL which which showed a comparable pattern of results. KT itself does not appear to be the main risk factor for the development of mental impairments.

## Introduction

Kidney Transplantation (KT) is considered the best treatment for chronic kidney disease (CKD) patients. In addition to the somatic benefits, such as reduced cardiovascular risk and overall mortality, patients also appear to benefit regarding individual health-related quality of life (HRQoL). HRQoL describes the subjective assessment of the impact of disease and its treatment across the physical, psychological and social domains of functioning and well-being [1]. The factors of HRQoL include satisfaction with life and individual happiness, as well as objective assessments of physical and psychological functioning [2–5]. Psychiatric research has predominantly focused on depressive disorders in non-transplanted CKD patients, in whom increased levels of preexisting depression and/or anxiety symptoms as well as a low level of social support have been found to be associated with decreased HRQoL. Impairments within this complex are simultaneously linked to kidney function, medication and treatment adherence and overall morbidity but differences in cadaveric and living donor kidney-transplanted patients were reported [6–18]. Prevalence rates of 25% for depressive disorders are reported for CKD patients on dialysis [14–16, 19–22]. These numbers are considerably increased compared with those of the general population, in which prevalence rates of 6.9% are typical, and even cancer patients with mean prevalence rates up to 17% [9, 23, 24].

Much less is known about the prevalence of depression and anxiety and the corresponding associations with HRQoL in transplanted CKD (CKD-T) patients [13, 22, 25]. In KT, mean prevalence rates of depressive diseases of 25% were found, even with a functioning graft [22, 25]. Single studies report extraordinary prevalence rates of up to 70% [23, 26–28]. Reduced kidney function, as well as a poor quality of life are suggested to be independently associated with an increased risk of mortality or earlier graft loss [11, 12, 22, 29–33]. Moreover, psychosocial factors, such as non-spousal status or female gender, were identified as risk factors. Unfortunately, these findings are limited because of the sample sizes, missing non-transplanted control groups or the retrospective character of data-analysis [33–35]. Most studies include both cadaveric and living donor kidney-transplanted patients without a differentiation between both groups. Some studies dealing with the group of living donor kidney-transplanted patients show tendencies of a better psychosocial and HRQoL-related outcome but are limited by small sample sizes or the retrospective design of the analyzes [36–39]. Furthermore, factors influencing the outcome such as medication regimen or former dialysis treatment are not included in all study protocols [18, 25].

The association of anxiety symptoms with the clinical course of CKD has received even less attention. Some studies found no elevated levels of anxiety in CKD, whereas other studies identified prevalence rates that ranged from 20–40% [13, 28, 34] as compared to rates up to 10% in the general population dependent on sociodemographic and economic influence factors [23]. Importantly, the existence of psychiatric impairments, such as anxiety or depression, is associated with an increased risk of somatic impairment in KT patients [29, 35, 40–46].

We have therefore initiated the *Psychiatric Impairments in Kidney Transplantation* (*PI-KT*) study to compare two groups of patients with regard to mental status before and after KT applying a cross-sectional design and to investigate potential relations of patient-reported outcomes to sociodemographic or disease-related characteristics in both subgroups. The data analysis investigated the prevalence and extent of anxiety and depression symptoms before and after KT in both groups while also taking into account HRQoL, coping (i.e., way of coping with the disease and impairments caused by it) and resilience, as well as disease-related and sociodemographic characteristics and their associations with mental status. We inferentially compared both groups with respect to differences in coping styles and resilience structures. Other influence factors like parameters of kidney function as well as sociodemographic characteristics were included within the data analysis.

## Methods and Participants

### Participants

The *PI-KT* study is a cross-sectional study that includes CKD/pre-KT and CKD-T patients treated in the Department of Nephrology. All CKD-T patients were cadaveric donor-transplanted. The study was approved by the ethics committee of the University of Erlangen-Nuremberg. All participating patients signed a written informed consent form, which included information regarding the study design and aims. Prevalent CKD/pre-KT patients on the waiting list for kidney transplantation and patients after KT were asked for their participation and data acquisition was performed over a 3-year time period (2010–2013). KT-patients getting their first (immediately after KT), three-month and twelve-month-protocol, also as indication-biopsies were enrolled. All patients were included at one visit. Those patients who had previously been transplanted and were awaiting a further kidney transplantation or who refused to participate were excluded from the CKD/pre-KT CKD-T comparisons.

### Procedure

Seventeen of 269 patients (5.0%) were excluded from the current analysis because of missing data so data of 252 patients (157/95 male/female, 101 (i.e. 40%) CKD/pre-KT and 151 (i.e. 60%) CKD-T) were analyzed (Table 1). In the CKD/pre-KT subgroup, there were 79 patients on dialysis, where 11 patients received peritoneal dialysis. 21 patients did not yet undergo dialysis treatment. In the CKD-T subgroup a total of 12 patients previously received peritoneal dialysis. To compare independent groups and to avoid inner-test bias through multiple test-assessments, we included only patient data that resulted from the first completion of the tests and attributed the patients to the pre-KT or CKD-T subgroups. Following the data acquisition, a multiple final controlled quality and error checking was performed. No systematic transcription errors were identified. In the initial step of the data analysis, the prevalence rates and the extent of depressive and/or anxiety symptoms were evaluated in a descriptive and inferential manner. We subsequently analyzed whether the psychological test scores differed between the pre-/and CKD-T group and whether there were relations among the test results that reflect mood (i.e. depressiveness and anxiety), resilience, coping strategies, and HRQOL and the parameters of kidney-functioning, as well as sociodemographic characteristics. The assessment consisted of a 45-minute diagnostic interview and psychological test questionnaires.

**Table 1:** Descriptive sample characteristics. Proportions are presented as valid percent.

	Total sample (N = 252)	CKD/Pre-KT (N = 101)	CKD-T (N = 151)	Test coefficient (effect size)	p-value
**Female**	95(37.7%)	33(32.7%)	62(41.1%)	χ^2^(1) = 1.81 (V = 0.09)	0.188
**Male**	157(62.3%)	68(67.3%)	89(58.9%)		
**Age (years), mean values**	51.6±14.3 (N = 251)	49.1±14.3 (N = 100)	53.3±14.1 (N = 151)	U = 6294.00, z = -2.23 (r = -0.14)	0.026
**Single/without partnership**	66(30.4%)	26(26.5%)	40(33.6%)	χ^2^(1) = 1.27 (V = 0.08)	0.300
**Married/ with partnership**	151(69.6%)	72(73.5%)	79 (66.4%)		
**Up to basic primary school**	49(22.5%)	19(19.4%)	30 (24.9%)		
**High School**	100(45.9%)	49(48.5%)	51(42.5%)	χ^2^(2) = 1.53 (V = 0.09)	0.471
**University-entrance diploma**	63(28.9%)	27(26.7%)	36(30.0%)		
**Duration of dialysis (months)**	58.0±55.6 (N = 193)	35.4±42.5(N = 76)	72.7±58.3(N = 117)	U = 2455.50, z = -5.25 (r = -0.38)	< 0.001*
**eGFR mL/min**	-	-	29.8±18.9(N = 123)		
**Creatinine mg/dL**	-	-	3.7±6.9(N = 124) ^#^		
**Leucocytes/10** ^**3**^ **μL**	7.7±3.5(N = 155)	7.5±3.0(N = 35)	7.8±3.6(N = 120)	U = 2085.00, z = -0.06 (r = -0.01)	0.950
**CRP mg/L**	9.9±16.5(N = 140)	4.5±5.2(N = 23)	11.0±17.7(N = 117)	U = 1121.00, z = -1.26 (r = -0.11)	0.210

^#^ Due to emergency patients with very high creatinine levels, median for creatinine is considerably lower with 2.1 mg/dL.

*p-value is equal to or remains below the critical p-value after Bonferroni-Holm adjustment for multiple inferential tests (total number of tests to see whether subgroups are comparable with respect to characteristics was N = 9).

### Sociodemographic characteristics and parameters of kidney-functioning

The sociodemographic characteristics, such as age, gender, marital status, and educational level, were collected from all patients (Table 1). Moreover, somatic information on cadaver-transplantation, type and duration of dialysis treatment, and laboratory findings, including leucocytes, C-reactive protein (CRP), estimated-glomerular filtration rate (e-GFR) and Serum-Creatinine (S-Creatinine), were recorded (Table 1).

### Patient-reported outcomes (PRO) and applied test assessments

To assess the prevalence rates and extent of depressive and/or anxiety symptoms, we used the **Hospital Anxiety and Depression Scale (HADS-D/A)** [47] (Table 2). The HADS is a validated screening instrument for the presence of depression and anxiety symptoms, which is widely used in hospital settings. It consists of 14 Likert scaled items with two subscales (0–21). Each subscale includes seven items scored on a four-point scale between zero and three. Subscale scores of up to 7 are considered normal, whereas scores of 8–10 are regarded as borderline and set as clinical manifestation. Both scales can be interpreted independently from each other [47].

**Table 2:** Comparisons of patient-reported outcomes between CKD/pre- and CKD-T patients. Comparison of patient-reported HADS-D/A, HRQoL, coping and resilience outcomes between pre-KT and CKD-T patients. Descriptive results are presented as arithmetic mean±standard deviation (median, interquartile range). Results of Mann-Whitney U-Test include test coefficients, effect sizes (r), and exact p-values. Values set in bold indicate p-values ≤ 0.05.

	CKD/Pre-KT	CKD-T	Test coefficients, (effect size r)	Exact p-values
**Depressive disturbances (HADS-D)**	4.91 ± 3.39 (4.00, IQR: 4.00)	4.48 ± 2.96 (4.00, IQR: 4.00)	U = 3968.50, z = -0.49 (-0.04)	0.624
**Anxiety symptoms (HADS-A)**	5.14 ± 3.74 (5.00, IQR: 5.00)	5.01 ± 4.04 (4.00, IQR: 6.00)	U = 3985.50, z = -0.44 (-0.03)	0.659
**Depressive coping (FKV scale 1)**	2.01 ± 0.70 (1.80, IQR: 1.20)	1.97 ± 0.64 (2.00, IQR: 1.00)	U = 3833.50, z = -0.16 (-0.01)	0.874
**Active, problem-focused coping (FKV scale 2)**	3.42 ± 0.80 (3.60, IQR: 1.00)	3.54 ±0.90 (3.80, IQR: 1.20)	U = 3473.00, z = -1.22 (-0.09)	0.225
**Diversion & self-encouragement (FKV scale 3)**	3.11 ± 0.72 (3.00, IQR: 1.00)	3.16 ± 0.88 (3.40, IQR: 1.20)	U = 3626.50, z = -0.77 (-0.06)	0.445
**Religiousness & search for coherence (FKV scale 4)**	2.71 ± 0.72 (2.60, IQR: 1.00)	2.93 ± 0.73 (3.00, IQR: 1.00)	**U = 3229.00, z = -2.02 (-0.15)**	**0.043**
**Trivializing & wishful thinking (FKV scale 5)**	2.09 ± 0.80 (2.00, IQR: 0.90)	2.10 ± 0.79 (2.00, IQR: 1.30)	U = 3835.00, z = -0.26 (-0.02)	0.796
**Resilience Acceptance Score (RS-A)**	45.37 ± 8.56 (48.00, IQR: 12.00)	45.43 ± 8.11 (47.00, IQR: 11.00)	U = 3946.00, z = -0.20 (-0.01)	0.845
**Resilience Competence Score (RS-C)**	98.88 ± 17.10 (103.00, IQR: 20.00)	97.64 ± 16.20 (100.00, IQR: 18.00)	U = 3682.50, z = -0.95 (-0.07)	0.344
**SF-12 PCS-Score**	49.39 ± 7.38 (48.59, IQR: 13.60)	43.89 ± 5.27 (44.69, IQR: 7.16)	**U = 405.00, z = -2.96 (-0.34)**	**0.003** *
**SF-12 MCS-Score**	43.70 ± 6.32 (43.84, IQR: 10.23)	45.94 ± 7.33 (46.51, IQR: 12.89)	U = 574.00, z = -1.15 (-0.13)	0.254

*p-value is equal to or remains below the critical p-value after Bonferroni-Holm adjustment for multiple inferential tests (i.e. the number of subscales of the corresponding patient-reported outcome tool).

#### Short Form 12-Item Health Survey (SF-12)—an indicator of health-related quality of life

The HRQoL concept defines a complex of various domain-like qualities, including physical and psychological functioning, physical, psychological and social domains of functioning and well-being. It includes medical aspects, such as physical functioning (e.g., ability to perform daily activities), mental functioning (e.g., emotional and mental well-being), social functioning (e.g., relationships with others and participation in social activities), and perception of health status, pain, and overall satisfaction with life. Because of its brevity and good performance in clinical use, the SF-12 has become a widespread measure of health status and the corresponding HRQoL [48, 49]. In the final step of the calculation, the 12 items are aggregated into two health summary scales that reflect physical (**PCS-12**) and mental (**MCS-12**) components, which range from 0 (worst) to 100 (best). Both summary scales have previously been used to evaluate physical and emotional well-being as important indicators of HRQoL in various samples, including CKD/pre-KT and KT patients [46, 50].

Resilience is defined as an individual’s ability to recover quickly from an illness, which enables the individual to resume his or her original shape or position after being affected by negative psychosocial influence factors [51–53]. There are defining attributes of resilience, such as rebounding reintegration/high expectancy, self-determination, positive relationships, social support, flexibility, and a sense of humor. These factors provide opportunities of communication and support; thus, they are important in view of their contribution to enhancements in quality of life [54]. The structure of the **Resilience Scale (RS)** represents two factors referred to as personal competence (**RS-C**) and acceptance (**RS-A**) of self and life. Positive correlations with adaptational outcomes (physical health, morale, and life satisfaction) and a negative correlation with depression support the construct validity of the RS. The significant outcomes or consequences of resilience are effective coping, mastery, and positive adaptation. Regardless of the degree of these consequences, their presence is a consistent outcome of the concept of resilience. Effective coping is best described as effectively managing the adversity one faces to function at an optimal level. Mastery is defined as possessing great skill or knowledge; and positive adaptation occurs when an individual is rebounding or recovering from a disruptive or adverse event and the recovery is beneficial or effective [55–58]. As previously noted, resilience is closely related to the concept of coping and the use of coping styles. Coping, which can be defined as the ability to manage and adjust external stress factors, is also mediated by other factors, such as the extent and severity of illness, treatment requirements, and the interindividual ability to cope and adjust [45, 59]. Coping styles can be broadly classified into two main approaches: problem focused, which involves the management or alteration of the problem with the environment that causes distress, and emotion focused, which involves the regulation of the emotional response to the problem using techniques, such as avoidance and denial. Individuals with a chronic illness often use both approaches, depending on the stressor and individual circumstances [42, 60, 61]. For the assessment of coping structures, the **Coping Self-Questionnaire (Freiburger Fragebogen zur Krankheitsverarbeitung, FKV in German)** was used. This is a questionnaire that comprises 102 items (12 scales) measured on 5-point Likert-scalesand asks patients for their opinion on how to properly cope with their illness. The FKV contains five subscales, which include depressive coping (FKV-scale 1), active, problem-focused coping (scale 2), diversion and self-encouragement (scale 3), religiousness & search for coherence (scale 4) and trivializing & wishful thinking (scale 5) [62].

### Statistical Data Analysis

The data were analyzed using SPSS version 21 (IBM Corp., Armonk, NY). In the initial step of the analysis, we investigated the demographic characteristics of the CKD/pre- and CKD-T patients (Table 1); in a subsequent step, we inferentially compared the PRO measures via non-parametric Mann-Whitney-U tests with exact p-values and corresponding effect sizes (Table 2). Chi-Square tests were used to compare the recently transplanted patients with the patients transplanted longer ago regarding the HADS-D/A scores. The latter analysis also extended to the prevalence and extent of anxiety and/or depressive symptoms (HADS-D/A) in the pre-KT and CKD-T subgroups. The set of psychological test tools was completed via measures of coping (FKV), resilience (RS) and HRQoL (SF-12), which enabled us to investigate their relations with sociodemographic and disease-related characteristics in both subgroups. Corresponding correlations are expressed as Spearman (r_s_) or pointbiserial correlation coefficients (r_pb_) (Tables 3 and 4). Adjustments for multiple inferential tests were implemented using Bonferroni-Holm adjustment for each tool separately; the strictest adjustment was applied to the lowest p-value followed by more liberal steps of adjustment for the remaining comparisons in an ascending order of p-values. The initial level of correction was equal to the conventional Bonferroni adjustment, i.e., the conventional type I error probability of 5% (p = 0.05) divided by the number of subscales obtained from the corresponding tool in each subgroup. Critical p-values for subsequent steps were obtained by dividing 5% by the number of remaining comparisons for each PRO. The descriptive data are presented as the mean ± standard deviation if not otherwise stated.

**Table 3:** Correlations of patient-reported outcomes with sociodemographic characteristics. Spearman rho correlation coefficients are presented for age and duration of dialysis while pointbiserial correlation coefficients are presented for gender and partnership. Additionally, p-values and corresponding sample size for each coefficient are presented. Values set in bold indicate p-values ≤ 0.05.

	Age		Gender		Partnership (yes /no)		Duration of dialysis	
	CKD/pre-KT	CKD-T	CKD/pre-KT	CKD-T	CKD/pre-KT	CKD-T	CKD/pre-KT	CKD-T
**Depressive disturbances (HADS-D)**	0.10 (0.383) *N* = 74	0.02 (0.834) *N* = 112	0.12 (0.329) *N* = 74	-0.05 (0.568) *N* = 112	-0.14 (0.242) *N* = 74	-0.28 (0.003)*N = 111	-0.03 (0.825) *N* = 55	0.06 (0.568) *N* = 104
**Anxiety symptoms (HADS-A)**	0.22 (0.056) *N* = 74	0.00 (0.981) *N* = 112	0.06 (0.636) *N* = 74	0.00 (0.947) *N* = 112	-0.12 (0.329) *N* = 74	-0.17 (0.082) *N* = 111	0.00 (0.982) *N* = 55	0.17 (0.078) *N* = 104
**Depressive coping (FKV scale 1)**	0.09 (0.431) *N* = 72	-0.10 (0.328) *N* = 108	**0.27 (0.023) *N* = 72**	0.09 (0.342) *N* = 108	-0.17 (0.159) *N* = 72	-0.12 (0.233) *N* = 107	-0.07 (0.622) *N* = 54	0.08 (0.413) *N* = 100
**Active, problem-focused coping (FKV scale 2)**	0.30 (0.010)*N = 72	0.08 (0.387) *N* = 108	-0.12 (0.318) *N* = 72	0.08 (0.425) *N* = 108	**0.25 (0.033) N = 72**	0.14 (0.146) *N* = 107	-0.26 (0.060) *N* = 54	-0.12 (0.254) *N* = 100
**Diversion & self-encourage-ment (FKV scale 3)**	-0.01 (0.927) *N* = 72	-0.06 (0.556) *N* = 108	-0.01 (0.968) *N* = 72	0.06 (0.573) *N* = 108	0.08 (0.523) *N* = 72	0.04 (0.699) *N* = 107	-0.14 (0.331) *N* = 54	-0.05 (0.610) *N* = 100
**Religiousness & search for coherence (FKV scale 4)**	0.01 (0.955) *N* = 72	**0.22 (0.020) *N* = 109**	**0.25 (0.036) *N* = 72**	0.15 (0.11) *N* = 109	0.068 (0.572) *N* = 72	**0.21 (0.031) *N* = 108**	-0.13 (0.352) *N* = 54	0.08 (0.443) *N* = 101
**Trivializing & wishful thinking (FKV scale 5)**	0.09 (0.432) *N* = 72	0.18 (0.058) *N* = 109	0.14 (0.242) *N* = 72	-0.07 (0.503) *N* = 109	-0.15 (0.204) *N* = 72	-0.06 (0.533) *N* = 108	-0.08 (0.570) *N* = 54	0.17 (0.095) *N* = 101
**Resilience Acceptance Score (RS-A)**	0.09 (0.448) *N* = 73	0.04 (0.680) *N* = 110	-0.05 (0.655) *N* = 73	0.01 (0.922) *N* = 110	0.36 (0.002)*N = 73	0.12 (0.200) *N* = 110	-0.14 (0.328) *N* = 54	-0.12 (0.218) *N* = 102
**Resilience Competence Score (RS-C)**	0.31 (0.009)* N = 73	-0.03 (0.739) *N* = 110	-0.09 (0.431) *N* = 73	-0.04 (0.658) *N* = 110	0.36 (0.002)*N = 73	0.02 (0.804) *N* = 110	**-0.30 (0.030) *N* = 54**	-0.15 (0.127) *N* = 102
**SF-12 PCS-Score**	-0.09 (0.635) *N* = 29	0.02 (0.893) *N* = 47	-0.20 (0.291) *N* = 29	0.22 (0.132) *N* = 47	-0.11 (0.583) *N* = 29	**-0.31 (0.038) *N* = 47**	0.17 (0.460) *N* = 21	**0.34 (0.026) *N* = 42**
**SF-12 MCS-Score**	0.02 (0.915) *N* = 29	0.07 (0.665) *N* = 47	-0.01 (0.958) *N* = 29	-0.05 (0.752) *N* = 47	0.12 (0.534) *N* = 29	0.05 (0.733) *N* = 47	-0.09 (0.703) *N* = 21	-0.27 (0.083) *N* = 42

*p-value is equal to or remains below the critical p-value after Bonferroni-Holm adjustment for multiple inferential tests (i.e. the number of subscales of the corresponding patient-reported outcome).

**Table 4:** Relations of patient-reported outcomes to disease-related parameters of kidney-functioning. Spearman rho correlation coefficients (p-values) are presented, including corresponding sample size for coefficient calculation. Values set in bold indicate p-values ≤ 0.05. For correlations of SF-12 and s-creatinine in the CKD/pre-KT subgroup without dialysis, we did not present results due to the sample size being below 5.

	S-creatinine	eGFR		CRP		Leukocytes	
	CKD/pre-KT (excl. dialysis)	CKD/pre-KT (excl. dialysis)	CKD-T	CKD/pre-KT (incl. dialysis)	CKD-T	CKD/pre-KT (incl. dialysis)	CKD-T
**Depressive disturbances (HADS-D)**	**-0.79 (0.033)** * ***N* = 7**	0.75 (0.054) *N* = 7	0.05 (0.613) *N* = 110	-0.37 (0.187) *N* = 14	0.00 (0.976) *N* = 104	-0.21 (0.361) *N* = 22	0.08 (0.389) *N* = 106
**Anxiety symptoms (HADS-A)**	**-0.88 (0.008)** * ***N* = 7**	**0.85 (0.017)** * ***N* = 7**	0.04 (0.676) *N* = 110	-0.10 (0.731) *N* = 14	0.01 (0.942) *N* = 104	0.04 (0.861) *N* = 22	0.03 (0.761) *N* = 106
**Depressive coping (FKV scale 1)**	-0.32 (0.491) *N* = 7	0.27 (0.561) *N* = 7	0.00 (0.984) *N* = 106	-0.54 (0.055) *N* = 13	-0.01 (0.931) *N* = 100	0.02 (0.933) *N* = 21	0.10 (0.337) *N* = 102
**Active, problem-focused coping (FKV scale 2)**	0.05 (0.908) *N* = 7	-0.06 (0.908) *N* = 7	-0.08 (0.398) *N* = 106	0.35 (0.240) *N* = 13	0.02 (0.813) *N* = 100	-0.05 (0.844) *N* = 21	-0.07 (0.478) *N* = 102
**Diversion & self-encourage-ment (FKV scale 3)**	0.25 (0.585) *N* = 7	-0.23 (0.624) *N* = 7	0.05 (0.622) *N* = 106	-0.16 (0.601) *N* = 13	-0.04 (0.680) *N* = 100	-0.07 (0.760) *N* = 21	-0.05 (0.653) *N* = 102
**Religiousness & search for coherence (FKV scale 4)**	0.18 (0.699) *N* = 7	-0.21 (0.653) *N* = 7	0.05 (0.584) *N* = 107	-0.28 (0.352) *N* = 13	0.07 (0.509) *N* = 101	0.11 (0.640) *N* = 21	-0.17 (0.091) *N* = 103
**Trivializing & wishful thinking (FKV scale 5)**	-0.73 (0.064) *N* = 7	0.67 (0.100) *N* = 7	0.03 (0.735) *N* = 107	0.22 (0.471) *N* = 13	-0.18 (0.079) *N* = 101	0.041 (0.860) *N* = 21	0.09 (0.359) *N* = 103
**Resilience Acceptance Score (RS-A)**	0.74 (0.057) *N* = 7	-0.69 (0.085) *N* = 7	0.07 (0.481) *N* = 108	0.44 (0.112) *N* = 14	0.15 (0.132) *N* = 102	0.21 (0.345) *N* = 22	-0.04 (0.666) *N* = 104
**Resilience Competence Score (RS-C)**	**0.96 (< 0.001)** * ***N* = 7**	**-0.96 (0.001)** * ***N* = 7**	0.12 (0.208) *N* = 108	0.22 (0.458) *N* = 14	0.19 (0.053) *N* = 102	0.23 (0.310) *N* = 22	0.07 (0.463) *N* = 104
**SF-12 PCS-Score**	—-	—-	-0.12 (0.412) *N* = 46	0.18 (0.613) *N* = 10	0.15 (0.344) *N* = 43	-0.20 (0.505) *N* = 13	-0.13 (0.428) *N* = 42
**SF-12 MCS-Score**	—-	—-	-0.03 (0.848) *N* = 46	0.17 (0.649) *N* = 10	0.05 (0.769) *N* = 43	0.00 (1.000) *N* = 13	0.21 (0.190) *N* = 42

*p-value is equal to or remains below the critical p-value after Bonferroni-Holm adjustment for multiple inferential tests (i.e. the number of subscales of the corresponding patient-reported outcome tool).

## Results

### Anxiety and depressive symptoms

In the CKD/pre-KT subgroup, 23.0% of the patients exhibited increased HADS-D scores and 21.6% of the patients exhibited increased HADS-A scores (i.e., subscale scores of ≥ 8), whereas in the CKD-T subgroup, elevated levels were found in 13.4% (HADS-D) and 25.0% (HADS-A) of the patients, respectively. However, a comparison of the frequency of increased depression and anxiety scores between the CKD/pre-KT and CKD-T patients did not indicate any differences in view of depressive symptoms (χ^2^_Depression_(1) = 2.87, p = 0.11) or anxiety (χ^2^_Anxiety_(1) = 0.28, p = 0.60); this finding was confirmed in view of the average HADS-A/HADS-D scores (Table 2). The average HADS-D scores were 4.91±3.39 (CKD/pre-KT) and 4.48±2.96 (CKD-T) (Table 2). Slightly higher scores were obtained for anxiety: HADS-A 5.14±3.74 (CKD/pre-KT) and 5.01±4.04 (CKD-T).

The prevalence rates for depressive and anxiety symptoms were not significantly different within the subgroups of the CKD-T patients (χ^2^_Depression_(2) = 0.06, p = 1.00; χ^2^_Anxiety_(2) = 1.83, p = 0.42) when considering the following of timeframes: 0–7 days after KT, 8–90 days after KT, and more than 91 days after KT.

Increased depressive symptoms were correlated with living alone in the CKD-T group (r_pb_ = -0.28; p = 0.003), which also held true after adjustment for multiple testing. However, the effect size of this relation was small. No further relation was identified between anxiety and depressive symptom severity and sociodemographic characteristics (Table 3). According to the results obtained by the Spearman correlational analysis, the HADS scores for anxiety and depressive symptoms were significantly related to S-creatinine (r_S(HADS-D)_ = -0.79, p = 0.033; r_S(HADS-A)_ = -0.88, p = 0.008) in the CKD/pre-KT patients not on dialysis (Table 4). The corresponding effect sizes of these coefficients were large. Thus, scores that indicated higher levels of anxiety and depressiveness appear to be accompanied by lower levels of S-creatinine prior to but not after transplantation.

### Coping and resilience

Both subgroups of patients exhibited the same pattern of results in the evaluation of appropriate strategies to cope with the impact of their disease with active, problem-focused strategies considered most feasible (CKD/pre-KT: 3.42±0.80, CKD-T: 3.54±0.90) followed by diversion and self-encouragement (CKD/pre-KT: 3.11±0.72, CKD-T: 3.16±0.88), and religiousness and search for coherence (CKD/pre-KT: 2.71±0.72, CKD-T: 2.93±0.73). Trivializing and wishful thinking (CKD/pre-KT: 2.09±0.80, CKD-T: 2.10±0.79), as well as depressive coping strategies (CKD/pre-KT: 2.01±0.70, CKD-T: 1.97±0.64) were considered least appropriate. However, even the strategies considered most appropriate received maximum average scores of only 3.5 on a scale with a maximum value of 5. Inferential comparisons of the FKV subscales indicated no differences after the adjustment for multiple testing. Religiousness and search for coherence exhibited an indication for higher values in the CKD-T subgroup, which did not remain significant after a Bonferroni-Holm adjustment (adjusted level of significance for the strongest FKV result: p ≤ 0.01). Spearman correlations for FKV coping strategies and sociodemographic characteristics indicated a number of tendencies with small effect sizes; however, the only significant finding had a medium effect size and indicated that the CKD/pre-KT patients had a more active, problem-focused point of view of coping with the challenges that result from the underlying disease with increased age (r_S_ = 0.30, p = 0.01). With respect to the parameters of kidney function, we could not identify a significant relation with coping strategies after adjustment. However, an interesting pattern of results could be observed: Regarding the effect sizes of the coefficients in the CKD/pre-KT group, there were a number of medium (r = 0.3–0.49) to large (r ≥ 0.5) effects, which indicate relations of depressive coping, active coping and trivializing/wishful thinking to S-creatinine, as well as, in part, to CRP (Table 4); this pattern of results was not identified in the CKD-T patients.

The resilience scores for acceptance (CKD/pre-KT: 45.37±8.56, CKD-T: 45.43±8.11) and competence (CKD/pre-KT: 98.88±17.10, CKD-T: 97.64±16.20) were not different between the two subgroups. In the CKD/pre-KT patients, medium correlation coefficients were identified between the acceptance subscale and the presence of a partner (r_pb_ = 0.36, p = 0.002), as well as between the competence subscale and age (r_S_ = 0.31, p = 0.009) and partnership status (r_pb_ = 0.36, p = 0.002). Furthermore, higher values on the competence scale also appear to be related to a shorter duration of dialysis (r_S_ = -0.3, p = 0.03); however, this finding did not remain significant after adjustment (adjusted level of significance for the strongest FKV result: p ≤ 0.025). Interestingly, higher values on both resilience scales also appear to be related to higher S-creatinine levels in the CKD/pre-KT patients without dialysis (r_S(RS-A)_ = 0.74, p = 0.057; r_S(RS-C)_ = 0.96, p<0.001), which also support the results observed for the HADS. Although the correlation coefficient for the acceptance scale failed to reach significance because of the small sample size for this calculation, it indicates a considerable effect with a coefficient that exceeds r = 0.5. Again, these results were identified only in CKD/pre-KT patients.

### Health-related quality of life

In both subgroups, the SF-12 physical (CKD/pre-KT: 49.39±7.38, CKD-T: 43.89±5.27, normal control sample 49.60) and mental summary (CKD/pre-KT: 43.70±6.32, CKD-T: 45.94±7.33, normal control sample 52.30) scores indicated considerable impairment of HRQoL with mean scores below the center of the scale (i.e., a score of 50). In view of the physical HRQoL, the CKD/pre-KT patients were significantly different from the CKD-T subgroup on the PCS-12 (U = 405.00, z = -2.96, p = 0.003), which suggests the CKD/pre-KT patients felt mildly more comfortable, although on a low level. After adjustment, there were no significant relations between the HRQoL and the demographic characteristics or disease-related serum markers; however, there were medium effect coefficients, which suggest physical well-being is related to partnership status and duration of dialysis.

## Discussion

The major aims of the *PI-KT* study were to compare the prevalence rates of depression and anxiety symptoms in CKD/pre-KT vs. CKD-T patients in a prospective cross-sectional study design and to further assess both groups regarding coping, resilience and HRQoL. Additionally, the relationships between mental symptoms and somatic parameters of organ-function were examined. These data help to provide information for the identification of risk groups during the course of kidney transplantation.

It is a surprising finding that both the CKD/pre-KT and CKD-T groups exhibit similar results with regard to the occurrence and average severity of depressive symptoms. The levels of depressive symptoms are higher than in other somatic disease groups and therefore note both CKD/pre-KT and KT patients as an outstanding risk group for psychiatric comorbidities [23–25]. In CKD/pre-KT and CKD-T, the previously reported rates of depression vary but indicate a lower prevalence of psychiatric symptoms in transplanted individuals [5, 9, 63]. The present results suggest that CKD patients represent a stable risk group for mental impairments even after KT. These findings also emphasize the need for a continuous psychiatric supervision of CKD-T individuals.

According to our findings, the occurrence of anxiety also did not differ between the CKD/pre-KT and CKD-T patients. These results are in contrast to former findings that indicated lower rates of anxiety after KT [63, 64]. The question arises as to whether these findings are specific for CKD-T patients because of the fear of losing the graft or the anxiety induced by medical treatment, in particular immunosuppression after KT [63, 65, 66]. Because anxiety is cross-linked to depressive diseases and a risk factor for other mental and somatic diseases, it is advisable to use (short) anxiety assessments more often within the treatment of KT.

It may be expected that the prevalence rates of depression and anxiety are most pronounced immediately after KT. The tests for potential dependency of depressive/anxiety symptoms over time in the CKD-T group yielded no significant differences between the subgroups at the different time intervals after transplantation (0–7, 8–90, >90 days after KT). Increased and constant prevalence scores for depressiveness/anxiety over time CKD-T are remarkable because depression is a strong predictor of diminished outcomes after KT [10, 13]. In that context the limitation of analyzing two different patient groups via a cross-sectional design before and after KT has to be mentioned. It is also a limitation of our study only having analyzed a group of cadaveric-transplanted individuals. Accordingly, potentially significant differences in cadaveric vs. living donor kidney-transplanted patients cannot be excluded.

Sociodemographic influences on depressive and anxiety symptoms could be identified only with regard to living alone in the CKD-T group, which is only partially consistent with previous examinations [5, 33, 34, 67]. Gender-specific factors could not be identified in our examinations. This finding appears to extenuate the importance of sociodemograhic influences on the course of depression and anxiety in CKD.

Regarding coping structures and sociodemographic characteristics, only a higher age appears to be moderately related to a more active, problem-focused coping style after correction for multiple testing. This finding may be explained by older patients who have the possibility to choose from a larger variety of solutions to challenges they have faced. Initially, the mental reaction of getting an organ does not appear to substantially influence coping or HRQoL/resilience structures. Coping styles and resilience structures may change later within the course of KT because personality-associated and stable intrapsychic factors are responsible for these findings [57, 58].

These findings support future studies that compare patients with longtime grafts with freshly transplanted individuals, including specific personality-associated and structured interviews (e.g., SKID-I and SKID-II) to differentiate between stable and fluid or personality-associated factors. This approach would also include physician-based ratings instead of only self-ratings, such as in this study.

It is remarkable that after the correction for intrapsychic factors, age-dependent sickness behavior persists. Thus, a higher age is correlated with competence structures, and a higher age in combination with partnership is associated with both competence and acceptance of the disease in CKD/-pre-KT. Therefore, these factors act similar to protective factors for mental impairments in CKD/pre-KT. A limitation of our examination is that potential therapy-related symptoms and side effects of medication, such as sleep disturbances or fatigue, may also have an impact on the HRQoL and depression trajectories evaluated in this data analysis.

Lower S-creatinine levels appear to be accompanied by higher anxiety and depressiveness in patients prior to but not after KT. Remarkably, this effect is still maintained and even more pronounced with respect to anxiety when patients on dialysis are excluded; however, the resulting sample size is small in this context. An explanation for this finding could be found in the process of accepting the diagnosis with CKD and facing the challenges that result from disease-related impairments. Thus, this subgroup of patients may still exhibit good kidney functioning in view of S-creatinine levels, whereas this new situation is a challenge they still need to cope with, which could in turn increase the HADS-scores [3, 9, 68].

However, it is remarkable that regarding the effect sizes in the CKD/pre-KT subgroup, there are a number of medium (r = 0.3–0.49) to large (r ≥ 0.5) effects, which indicates relations among depressive coping, active coping and trivializing/wishful thinking and S-creatinine, as well as partly CRP (Table 3). This pattern was not identified in the KT patients, even though several KT patients had increased S-creatinine or CRP levels. Therefore, increased S-creatinine may be a risk factor for mental disturbances in CKD/pre-KT.

## Conclusions

Taken together, the present study indicates that prevalence of depressiveness/anxiety, resilience/coping structures and HRQoL remain surprisingly constant in patients before and after kidney transplantation. Therefore, the burden of KT itself does not appear to be the main risk factor for the development of mental impairments CKD-T. To better identify potentially causative constant factors that are somatic and related to the underlying disease or psychosocial in nature, further longitudinal studies are necessary.
